# Efficacy and safety of non-invasive brain stimulation techniques for the treatment of nicotine addiction: A systematic review of randomized controlled trials

**DOI:** 10.3934/Neuroscience.2024014

**Published:** 2024-07-01

**Authors:** Fiammetta Iannuzzo, Silvia Crudo, Gianpaolo Antonio Basile, Fortunato Battaglia, Carmenrita Infortuna, Maria Rosaria Anna Muscatello, Antonio Bruno

**Affiliations:** 1 Department of Biomedical and Dental Sciences and Morphofunctional Imaging, University of Messina, Via Consolare Valeria 1, Contesse, Messina 98125, Italy; 2 Department of Medical Sciences, Neurology and Psychiatry, Hackensack Meridian School of Medicine, Nutley, NJ 07110, USA; 3 Psychiatry Unit, Polyclinic Hospital University of Messina, Via Consolare Valeria 1, Contesse, 98125 Messina, Italy

**Keywords:** NIBS, TMS, tDCS, nicotine addiction, neuromodulation

## Abstract

Non-Invasive Brain Stimulation (NIBS) techniques seem to be effective in treating tobacco use disorder. We aimed to analyze what kinds of protocols are used to treat nicotine addiction in term of cessation and/or reduction and to evaluate the long-term effects of NIBS techniques.

We searched PubMed, Scopus, and Web of Science for papers published, with combinations of the following search terms: “*Non-invasive brain stimulation OR TMS OR transcranial magnetic stimulation OR tDCS OR transcranial direct current stimulation OR transcranial electrical stimulation OR TES AND Nicotine addiction*”.

We conducted a preliminary search, which revealed papers on the topic. Articles were included in the review according to the following inclusion criteria: English language, publication in peer reviewed journals, articles about studies performed on non-invasive brain stimulations techniques, and RCT studies. Studies involving clinical populations with organic or psychiatric diseases were excluded. We found 280 articles. Of these, at the first screening and conducted by title and abstract, 63 studies were excluded after duplicates were removed (118). After the second screening conducted by full-text examination, 45 articles were excluded. Ten studies met the inclusion criteria and were included in the review.

The clinical benefits of NIBS, including the fast onset and minor side effects, showed that this kind of treatment could be helpful in patients with a long history of smoking in terms of cessation and abstinence rates.

## 1. Introduction

The severe health and economic consequences of the global tobacco use made tobacco control an essential public health priority [1]. Cigarette smoking has been responsible for more than 200 million deaths over the past 30 years, since it is one of the most important risk factors for premature mortality and morbidity globally. Smoking is a major cause of cardiovascular morbidity and mortality worldwide [2] and it is the cause of many other diseases such as COPD and lung cancer [3],[4]. Therefore, reducing the prevalence of smoking is probably the most effective and cost-effective form of prevention of disease, disability and death, as well as a key public health priority [1],[5]. Moreover, tobacco smoking is the most common substance-use disorder, characterized by craving, withdrawal, and compulsive use despite negative consequences [6]. Numerous lines of research have highlighted the addictive nature of cigarette smoking through the nicotine action on reward systems. The rewarding properties of nicotine that promote drug intake involve the mesolimbic projection of dopamine from the ventral tegmental area to the nucleus accumbens, while the aversive properties of nicotine, which limit drug intake and mitigate withdrawal symptoms, involve the projection of the fasciculus retroflexus from the medial habenula to the interpeduncular nucleus. Additional brain regions have also been implicated in various aspects of nicotine dependence, such as prefrontal cortex (PFC), ventral striatum, ventral pallidum, nucleus tractus solitarius, and insula [7]. All these brain regions are, directly or indirectly, interconnected, being part of an integrative, topographically organized cortico-striatal circuitry involved in goal-directed behavior and stimulus-value attribution, which is thought to play a crucial role in drug-seeking and drug-taking behavior [8]. Such mechanisms cause most people who attempt to quit smoking to experience craving symptoms, withdrawal symptoms, and fail the attempt, with only 3–10% having positive results after one year. Available treatments, such as behavioral support, varenicline, bupropione, and nicotine replacement therapy (NRT) improve the chances of these attempts. However, long term outcomes are relatively low, therefore, there is a need to identify new, effective, and safe alternatives to treat cigarette smoking addiction [9].

In this context, Non-Invasive Brain Stimulation techniques (NIBS), as transcranial magnetic stimulation (TMS) and transcranial direct current stimulation (tDCS), seems to enter into the new, innovative, and experimental therapies due to the advantages related to safety, tolerability, cost-effectiveness, and compatibility with other possible treatments.

Transcranial magnetic stimulation (TMS) is a tool that manipulates reward-related circuities during withdrawal correlates with levels of craving, relapse and continued nicotine consumption. TMS exploits a high-intensity magnetic field, generated by a light electric current in a coil, which when applied to the scalp, allows it to interfere with normal neural activity, modulating excitability and neuronal communication. Methods of TMS administration include conventional TMS, refers to the standard method of administering TMS therapy, usually using repeated delivery of magnetic pulses over time (rTMS), theta burst stimulation (TBS), a TMS protocol that delivers short bursts of magnetic pulses at a high frequency, and deep TMS (dTMS), using a specialized coil design that allows for stimulation of deeper brain structures compared to conventional TMS. The possibility of examining changes in cortical excitability after prolonged exposure to substances has given considerable impetus to the study of this technique in the field of addiction, proposing it as a therapy also in nicotine addiction. In this field, TMS is a non-invasive therapeutic practice which seems to be effective in reducing nicotine addiction [10].

Transcranial direct current stimulation (tDCS) is a neurostimulation technique based on the passage of a weak current (1–2 mA) across the cortex using at least two electrodes [11]. Effects of tDCS is due to the modification of the conductivity of sodium and calcium' channels and to the shifting of electrical gradients that affect the ion balance inside and outside the neuronal membrane, modulating its activation threshold.

Our objective is to analyze what kinds of protocols are used to treat nicotine addiction in terms of smoking cessation and/or reduction.

## 2. Materials and methods

This systematic review was reported in accordance with the 2020 Preferred Reporting Items for Systematic Reviews and Meta-Analyses (PRISMA) statement [12]. Our review protocol was pre-registered in April 2023 (PROSPERO registration number: CRD42023410083). No changes were made to the original protocol submitted on 27 March 2023.

### 2.1. Information sources and search strategy

We searched PubMed, Scopus and Web of Science for papers published for papers published between 1 January 2000 and 31 December 2023, with combinations of the following search terms: “*Non-invasive brain stimulation OR TMS OR transcranial magnetic stimulation OR tDCS OR transcranial direct current stimulation OR transcranial electrical stimulation OR TES AND Nicotine addiction*”.

### 2.2. Data extraction

We conducted a preliminary search, which revealed 280 papers. Articles were included in the review according to the following inclusion criteria: English language, publication in peer reviewed journals, articles about studies performed on non-invasive brain stimulations techniques, only RCT studies. Outcomes of interest are the effectiveness of noninvasive neuromodulation techniques used for the purpose of nicotine de-addiction. Studies involving clinical populations with organic or psychiatric diseases were excluded.

### 2.3. Data synthesis

**Figure 1.**
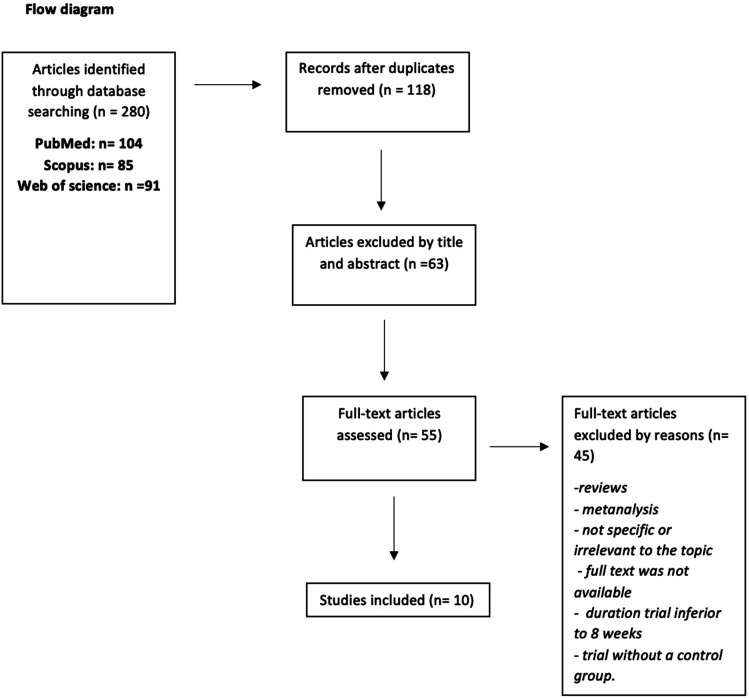
Flow diagram.

We found 280 articles. Of these, at the first screening and conducted by title and abstract, 63 studies were excluded after duplicates removed (n = 118). After the second screening conducted by full-text examination, 45 articles were excluded because they were reviews, metanalysis, not specific, irrelevant for the topic, or because the full text was not available, because the duration of the trial was inferior to 8 weeks or because the trial did not present a control group. Eventually, 10 studies met the inclusion criteria and were included in the review. The annexed table summarizes the selected articles (Table 1), whereas the annexed flow diagram (Figure 1) summarizes the selection process.

Two authors performed the initial search, independently reviewed, and selected the references based on the inclusion and exclusion criteria. The results were subsequently re-evaluated by a third author and the salient results were shown.

### 2.4. Risk of Bias

To assess the risk of bias in the findings, we used RoB version 2.0, the revised Cochrane risk of bias tool for randomized trials. RoB assesses the following domains: Randomization process, deviations from intended interventions, missing outcome data, measurement of the outcome, and selection of the reported result. As for study selection, two individuals independently estimated the risk of bias for each study. Disagreements has been resolved by consensus between the authors or by a third author. No significant publication bias was detected among the articles.

## 3. Results

Ten randomized controlled trials were identified from the literature search (see Table 1). Of these studies, 5 were in regards to treatment about conventional rTMS protocols, 1 study contained Theta Burst Stimulation, 3 studies were about deep TMS treatment, and 1 study was about tDCS treatment. The participants in the studies were cigarettes smokers, assessed through specific measures; they have been compared with subjects with nicotine addiction who underwent sham or other de-addiction techniques. Patients treated with NIBS had better outcomes compared with others.

**Table 1.** Selected articles.

**Authors**	**Number of participants**	**Stimulation technique**	**Brain target**	**Stimulation parameters**	**Trial duration**	**Control group**	**Follow-up**
Abdelrahman et al., 2021	62 (100% male/0%female)	HF-rTMS	L-DLPFC	80% MT 2000 pulses at 20 Hz	10 sessions (5 days per week)	Sham stimulation	3 months Lower cigarette consumption persisted
Amiaz et al., 2009	48 (44% male/56% female)	HF-rTMS	L-DLPFC	100% MT 50 pulses at 10 Hz	10 days	Sham stimulation	3 months Not sufficient data for high drop-out rates
Dieler et al., 2014	74 (54%/46% female)	iTBS	R-DLPFC	80% MT 600 Pulses at 50 Hz	3 weeks	Sham stimulation	3, 6 and 12 months increased abstinence rate
Dinur-Klein et al., 2014	115 (62% male/38% female)	Deep rTMS	R-L lateral PFC and insula	120% of MT HF:990pulses at 10 Hz. LF: 600pulses at 1 Hz	10 sessions (5 days per week) + 3 non-consecutive treatments.	Sham stimulation	6 months the reduction in cigarette consumption
Ghorbani Behnam et al., 2019	140 in NIBS groups and 35 in bupropion group (100%male /0% female)	tDCS	Anode F3 & Cathode F4	2 mA for 20 min	Longer: 2 weeks of daily stimulation + 10 weeks weekly booster Shorter: 20 sessions over 4 weeks	Sham stimulation And Bupropion group	6 months Failure in complete smoking cessation
Ibrahim et al., 2023	42 (n =24 active group; n =18 sham group)	Deep TMS	Insula	120% MT 1020 pulse at 10 Hz	for 20 sessions for four consecutive weeks (5 times/week)	Sham group; All participants received open label varenicline for 12 weeks	4 months High abstinence rates in active group.
Li et al., 2020	42 (45% male/55% female)	rTMS	L-DLPFC	100% MT 3000 pulses at 10 Hz	10 sessions (5 days per week)	Sham stimulation	3 months reduced cigarette consumption
Sheffer et al., 2018	29 (59%male/41% female)	rTMS	L-DLPFC	110% MT 900 pulses 20 Hz	8 sessions (4 days per week)	Sham stimulation	12 weeks the quit date CCA:66.7%
Trojak et al., 2015	37 (54%male/46% female)	rTMS	R-DLPFC	120% MT 6 trains of 60 pulses at 1 Hz	2-week of rTMS and NRT + 4-week of NRT alone	Sham stimulation	3 months maintained continuous abstinence
Zangen et al., 2021	262	Deep rTMS	R-L lateral PFC and insula	120% MT 1800 pulses at 10 Hz	3 weeks	Sham stimulation	18 weeks

### 3.1. rTMS

Five studies regarded conventional rTMS treatments.

In Abdelrahman et al. [13], 62 male smokers were randomized to receive TMS treatment for 10 sessions (5 days per week) with 3 months follow up. The aim was the evaluation of high frequency (20 Hz) repetitive transcranial magnetic stimulation (HF-rTMS) over the left dorsolateral prefrontal cortex (L-DLPFC) on nicotine-dependent cigarette smokers. Compared with sham treatment, active rTMS led to a larger reduction in the number of cigarettes/day; tobacco craving and nicotine dependence as well as depression and anxiety. In Amiaz et al. [14], 40 non-clinical smokers were randomized to receive 10 days of rTMS (10 Hz) on the left DLPFC vs sham treatment (20 patients for group). Cue provocation, i.e. exposure of the patients to smoking-related stimuli, was administered both in the active and sham group during treatment. The study showed a significant reduction in general nicotine craving between the first and 10th treatments although, no sufficient data about follow up were reported. The trial by Li et al. [15] evaluated the efficacy of 10-session daily of active high frequency (10 Hz) rTMS over the L-DLPFC coupled with cue provocation on smoking cessation on a small sample (n = 42) of smokers showing reduction in cigarette consumption compared to the sham and a high quit rate after 3 months.

Sheffer et al. [16] evaluated the effect of 8 session of high frequency rTMS (20 Hz) on L-DLPFC combined with the 8 evidence-based self-help relapse prevention booklets on decreasing delay discounting. A sample of 29 smokers, abstinent from 24 hours, was randomized to sham vs active treatment. No cue provocation was employed, and the patients were reading the relapse prevention booklets during the treatment. The treatment decreased delay discounting and increased latency to relapse, abstinence rates, and intervention uptake; abstinence was maintained up to 12 weeks after the last session.

Finally, in Trojak et al. [17], 37 smokers were randomized to receive inhibitory treatments, some were randomized to receive low frequency rTMS (1 Hz) over the right DLPFC, which was administrated in combination with NRT (nicotine replacement therapy) vs NRT only, to attenuate nicotine withdrawal symptoms, and reduce abstinence. The results showed that the active rTMS group had maintained continuous abstinence at the end of the treatment (2 weeks), while the follow up at weeks 6 and 12 was not adequately analyzed because of the large number of drop-outs at week 6 and 12.

### 3.2. Theta Burst TMS

The only study about theta burst TMS for smoking cessation [18] is a pilot study showing the effects of 4 sessions of intermittent theta burst stimulation (iTBS) on the right DLPFC as add-on treatment to cognitive behavior therapy (CBT) on nicotine craving and long-term abstinence. Seventy-two healthy smokers were randomly assigned to a treatment or a sham group. No differences in craving were reported. At the 3-months follow-up, the treatment group displayed an increased abstinence rate as compared to the sham group, but significant differences were not observed at the 6 and 12 months follow up.

### 3.3. Deep TMS

Researchers [19]–[21] evaluated effects of deep transcranial magnetic stimulation (dTMS) of the prefrontal cortex (PFC) and insula for smoking cessation. In the first study of Dinur-Klein et al., 115 subjects were assigned to three treatment arms: High-frequency (10 Hz), low-frequency (1 Hz), and sham deep TMS with and without smoking cue provocation. After 6 months, the reduction in cigarette consumption in the 10 Hz groups was significantly greater compared to the sham groups but not compared to the 1 Hz group. Zangen and colleagues, in a large multicentric study of deep TMS, recruited 262 chronic smokers meeting the DSM-V criteria for tobacco use disorder, and with at least one prior failed attempt to quit. Patients were randomly assigned to three weeks of bilateral dTMS of prefrontal cortex and insula with cue provocation vs sham. Researchers reported a significant difference in the primary outcome (continuous quit rate) between sham and control groups; in addition, a reduction in secondary outcome measures of consumption and craving was observed as early as two weeks into treatment.

Ibrahim et al. analyzed 42 participants that were randomized to receive either active (10 Hz) or sham rTMS targeting the insula, underlining how smokers in the active group had significantly higher abstinence rates than those in the sham group [23].

### 3.4. tDCS treatment

The study of Ghorbani et al. [22] evaluated abstinence rates at 6 months in subjects treated with two kinds of tDCS protocols: A longer duration tDCS protocol (20 sessions over 12 weeks: 2 weeks of daily stimulation followed by weekly booster sessions for 10 weeks) and a shorter stimulation protocol (20 sessions over 4 weeks), compared with subjects treated with sham or with bupropion. The results showed a higher success in bupropione group and long tDCS protocol compared to others, a failure in complete smoking cessation in short tDCS protocol, shams compared to bupropion group, and no statistically significant difference between groups with bupropion and long tDCS duration protocol.

### 3.5. Risk of bias

For all the studies under evaluation, risk of bias was calculated with a semi-automated tool for randomised trials. Overall, most of the studies (seven over nine) collected had high or medium risk of bias, with four studies over nine resulting in high risk of bias, and three studies over nine having some concerns. The most common concerns regarded bias due to deviation of the protocol from intended intervention and bias in the selection of the reported results. The results of semi-automated risk of bias evaluation are reported in Table 2.

**Table 2.** Risk of Bias evaluation.

	D1	D2	D3	D4	D5	Overall
Aberdam et al., 2021	+	+	+	+	-	-
Amiaz et al., 2009	-	-	+	+	+	-
Dieler et al., 2014	+	+	+	x	-	x
Dinur-Klein et al., 2014	+	+	+	x	-	x
Ghorbani-Behnam et al., 2019	+	-	+	+	+	-
Ibrahim et al., 2023	+	+	+	+	+	+
Li et al., 2020	+	+	+	+	+	+
Sheffer et al., 2018	+	+	+	+	+	+
Trojak et al., 2015	+	x	x	+	+	x
Zangen et al., 2021	+	x	+	+	x	x

Domains					Judgement
D1: Bias arising from the randomization process.		
D2: Bias due to deviations from intended intervention.					x high
D3: Bias due to missing outcome data.					- Some concerns
D4: Bias in measurement of the outcome.					+ Low
D5: Bias in selection of the reported result.		

## 4. Discussion

We analyze non-invasive brain stimulation interventions in reducing smoking and in maintenance of abstinence rates.

Our results show that NIBS techniques reduce the number of cigarettes smoked with good acceptability in all the studies, suggesting the potential efficacy of NIBS for smoking reduction when compared with sham treatment. These results are consistent with Tseng's results, which showed a significant effect on smoking reduction with NIBS versus sham [23]. In terms of smoking cessation, NIBS may improve smoking abstinence rates from 3 to 6 months after quitting smoking, compared with sham [24]. However, data show a great variability in NIBS techniques and in the parameters of stimulation used in the treatment of nicotine addiction.

In the analyzed rTMS protocols, high-frequency (10 Hz or 20 Hz) rTMS applied to the left DLPFC seems to be effective in decreasing smoking frequency in people with nicotine addiction. This datum is consistent with other studies [25] and suggest how high-frequency rTMS in left DLPFC may modulate neuroadaptation in the reward system. Previous studies underscored the melioration of craving in substance addiction with high-frequency TMS protocols over the right DLPFC, however, only in the trials of Trojack and Dieler the target area was the right DLPFC, although most of the evidence suggests that left DLPFC seems to be an appropriate target for treating nicotine dependence [26]. In any case, to this day, only a Level C recommendation has been proposed for the possible efficacy of HF rTMS of the left DLPFC in reducing cigarette consumption.

Studies showed a significant heterogeneity in terms of methods and patients' profile and they did not show an increase in long-term abstinence rate, especially in patients in comorbidity with psychiatric conditions [27].

Regarding deep TMS protocols, high frequency treatment for smoking cessation seems to be effective if compared to sham and to low frequency deep TMS protocols. Contrary to repetitive TMS, deep TMS employs complex 3-D coils to induce deep penetrating fields, thus guaranteeing more focal and selective stimulation in psychiatric disorders. For this reason, further studies could be needed to compare deep TMS to conventional TMS, to investigate if the deeper stimulation is more effective that the conventional in nicotine addiction.

TMS studies concur in stating that the DLPFC is an appropriate target area in the treatment of nicotine addiction; this is in line with the neurocognitive model of addiction, giving its important position in controlling cue-elicited drug craving and initiating drug abuse [28];on the other hand, there are no studies targeting other areas. The studies in which subcortical areas are considered agree on insula as the target area, which agrees with studies demonstrating insula as a promising target for brain stimulation [21].

Finally, the described tDCS protocol by Gorbahni et al. [22] explains anodal stimulation over the F3 region in combination with cathodal stimulation over the F4 region, showing that anodal stimulation over the left DLPFC provided higher treatment efficacy. This finding is consistent with studies that reported the efficacy of cathodal tDCS over the DLPFC on smoking intake compared with cathodal tDCS over other brain regions [29]; the precise mechanism responsible for the modulation remains unclear, although studies propose that tDCS may alter the activity or connectivity between the DLPFC and other prefrontal areas.

According to our literature review, no short duration protocols are considered in observed studies, as the shortest treatment period is 10 days. Drop-out rates seem to be similar in all the trials that ranging from 10 days to 3 weeks of treatment. For this reason, further studies are necessary to establish if shorter treatment (less than 10 days) could reduce dropout rates. In addition, there are no protocols consisting of combined NIBS techniques. According to our review, the studies that used combined strategies (a technique of neuromodulation together with another kind of treatment) did not show benefits for sustained abstinence [17],[18]; nevertheless, it could be useful to evaluate the possible efficacy and safety of new protocols consisting of combined NIBS techniques starting from the hypothesis that the combination of both techniques may be more effective and long-lasting than conventional protocols.

The present work has limitations. First, despite an effective search strategy of research, few studies were included. This may be explained by the relative novelty of the topic, as further investigations are required to ascertain the effects of NIBS on smoking and tobacco addiction. Most of the included studies have small sample sizes (only three studies counting more than 100 patients [19],[20],[22]. Among studies, risk of bias was relatively high and, therefore, the overall quality of the presented studies resulted low. In general, studies are affected by great heterogeneity, not only in terms of NIBS protocols used (treatment duration, stimulation site and parameters employed, use of cue provocation) but also in terms of baseline severity of smoking, outcome evaluation and duration of follow-up period. The major heterogeneity source among treatment protocols regarded rTMS protocols, for the choice of stimulation frequency (ranging between 10 and 20 Hz for high frequency protocols), the number of pulses and the stimulation intensity, expressed in terms of percentage of resting motor threshold (RMT). Heterogeneity in baseline severity was also particularly relevant, for study protocols employing cue provocation, which has been showed to have different effects on high-dependent versus low-dependent smokers [30]. Finally, only few studies employed smoking cessation as a primary outcome, while most of the investigations evaluated efficacy in terms of surrogate endpoint measures such as reduction of cigarette intake or decrease in measures of craving and addiction. Taken together, these limitations may reduce the interpretability of results; in general, care is recommended as further rigorous and high-quality investigations are required to ascertain the efficacy of NIBS techniques in reducing smoke-related behavior.

## 5. Conclusions

NIBS techniques seem to be safe and well-tolerated compared with other possible treatments. In analysed studies the results in terms of smoking reduction are better than the sham and equivalent in comparison with other interentions as bupropion. The rates of abstinence seem to persist for months although studies with 12 months follow up are needed to evaluate a long-term efficacy, since in most of the studies the evaluated follow-up is at 6 months.

The clinical benefits, including the fast onset and minor side effects, show that this kind of treatment could be helpful in patients with a long history of smoking and with several failed attempts to quit, using available options. Our data shows a sufficient safety of the use of these techniques according to the literature arguing how NIBS techniques are generally safe; despite the usual risks associated with the techniques that need to be screened prior to starting them [31].

However, the relative scarceness of rigorous, large-scale clinical trials and the great heterogeneity among investigators represent the stronger limitations to the interpretation of results. Further, high-quality studies are necessary to compare NIBS techniques with each other and to explore if the combination of more NIBS techniques may be more effective and long-lasting than a conventional protocols.

## 6. Future perspectives

Clinical application of neuromodulation techniques in nicotine addiction is a promising field of study that needs further investigation.

Whitin the field of neuromodulation, literature provides growing interest in developing personalized NIBS protocols based on individual characteristics to maximize therapeutic benefits and minimize side effects. Besides the need for a deeper understanding of these techniques, addressing the current challenges through rigorous research and technological advancements is essential for realizing the full potential of neuromodulation [32].

Congruently, NIBS techniques could benefit from the large use of artificial intelligence tools, for optimizing personalized medicine. The growing importance of machine learning (ML) in neuroscience research could find a correct use into the predictive models of NIBS protocols for smoking cessation by processing complex neuroimaging data to identify biomarkers linked to nicotine addiction and treatment responses [33]. ML models could forecast individual treatment outcomes by analyzing demographic, clinical, and neurobiological factors, allowing clinicians to customize NIBS personalized protocols and could enhance the precision and effectiveness of NIBS techniques by optimizing stimulation parameters and target regions using real-time neurofeedback.

The use of new investigative tools to identify patterns of brain structure associated with symptoms could contribute to our understanding of the structural and functional organization of the brain in neuropsychiatric disorders and could improve treatment based on neuromodulation.

## Use of AI tools declaration

The authors declare they have not used Artificial Intelligence (AI) tools in the creation of this article.

## References

[1] Reitsma MB, Kendrick PJ, Ababneh E (2021). Spatial, temporal, and demographic patterns in prevalence of smoking tobacco use and attributable disease burden in 204 countries and territories, 1990–2019: a systematic analysis from the Global Burden of Disease Study 2019. Lancet.

[2] Rezk-Hanna M, Benowitz NL (2019). Cardiovascular Effects of Hookah Smoking: Potential Implications for Cardiovascular Risk. Nicotine Tob Res.

[3] Bade BC, Dela Cruz CS (2020). Lung Cancer 2020. Clin Chest Med.

[4] Duffy SP, Criner GJ (2019). Chronic Obstructive Pulmonary Disease. Med Clin N Am.

[5] Le Foll B, Piper ME, Fowler CD (2022). Tobacco and nicotine use. Nat Rev Dis Primers.

[6] Potvin S, Tikàsz A, Dinh-Williams LL-A (2015). Cigarette Cravings, Impulsivity, and the Brain. Front Psychiatry.

[7] Goldstein RZ, Volkow ND (2011). Dysfunction of the prefrontal cortex in addiction: neuroimaging findings and clinical implications. Nat Rev Neurosci.

[8] Basile GA, Bertino S, Bramanti A (2021). Striatal topographical organization: Bridging the gap between molecules, connectivity and behavior. Eur J Histochem.

[9] Pipe AL, Evans W, Papadakis S (2022). Smoking cessation: health system challenges and opportunities. Tob Control.

[10] Rachid F (2016). Neurostimulation techniques in the treatment of nicotine dependence: A review. Am J Addict.

[11] Chase HW, Boudewyn MA, Carter CS (2020). Transcranial direct current stimulation: a roadmap for research, from mechanism of action to clinical implementation. Mol Psychiatry.

[12] Page MJ, McKenzie JE, Bossuyt PM (2021). The PRISMA 2020 statement: an updated guideline for reporting systematic reviews. BMJ.

[13] Abdelrahman AA, Noaman M, Fawzy M (2021). A double-blind randomized clinical trial of high frequency rTMS over the DLPFC on nicotine dependence, anxiety and depression. Sci Rep.

[14] Amiaz R, Levy D, Vainiger D (2009). Repeated high-frequency transcranial magnetic stimulation over the dorsolateral prefrontal cortex reduces cigarette craving and consumption. Addiction.

[15] Li X, Hartwell KJ, Henderson S (2020). Two weeks of image-guided left dorsolateral prefrontal cortex repetitive transcranial magnetic stimulation improves smoking cessation: A double-blind, sham-controlled, randomized clinical trial. Brain Stimul.

[16] Sheffer CE, Bickel WK, Brandon TH (2018). Preventing relapse to smoking with transcranial magnetic stimulation: Feasibility and potential efficacy. Drug Alcohol Depend.

[17] Trojak B, Meille V, Achab S (2015). Transcranial Magnetic Stimulation Combined With Nicotine Replacement Therapy for Smoking Cessation: A Randomized Controlled Trial. Brain Stimul.

[18] Dieler AC, Dresler T, Joachim K (2014). Can Intermittent Theta Burst Stimulation as Add-On to Psychotherapy Improve Nicotine Abstinence? Results from a Pilot Study. Eur Addict Res.

[19] Dinur-Klein L, Dannon P, Hadar A (2014). Smoking Cessation Induced by Deep Repetitive Transcranial Magnetic Stimulation of the Prefrontal and Insular Cortices: A Prospective, Randomized Controlled Trial. Biol Psychiatry.

[20] Zangen A, Moshe H, Martinez D (2021). Repetitive transcranial magnetic stimulation for smoking cessation: a pivotal multicenter double-blind randomized controlled trial. World Psychiatry.

[21] Ibrahim C, Tang VM, Blumberger DM (2023). Efficacy of insula deep repetitive transcranial magnetic stimulation combined with varenicline for smoking cessation: A randomized, double-blind, sham controlled trial. Brain Stimul.

[22] Ghorbani Behnam S, Mousavi SA, Emamian MH (2019). The effects of transcranial direct current stimulation compared to standard bupropion for the treatment of tobacco dependence: A randomized sham-controlled trial. Eur Psychiat.

[23] Tseng P, Jeng J, Zeng B (2022). Efficacy of non-invasive brain stimulation interventions in reducing smoking frequency in patients with nicotine dependence: a systematic review and network meta-analysis of randomized controlled trials. Addiction.

[24] Petit B, Dornier A, Meille V (2022). Non-invasive brain stimulation for smoking cessation: a systematic review and meta-analysis. Addiction.

[25] Lefaucheur J-P, Aleman A, Baeken C (2020). Evidence-based guidelines on the therapeutic use of repetitive transcranial magnetic stimulation (rTMS): An update (2014–2018). Clin Neurophysiol.

[26] Lefaucheur J-P, André-Obadia N, Antal A (2014). Evidence-based guidelines on the therapeutic use of repetitive transcranial magnetic stimulation (rTMS). Clin Neurophysiol.

[27] Mahoney JJ, Hanlon CA, Marshalek PJ (2020). Transcranial magnetic stimulation, deep brain stimulation, and other forms of neuromodulation for substance use disorders: Review of modalities and implications for treatment. J Neurol Sci.

[28] Liu Q, Yuan T (2021). Noninvasive brain stimulation of addiction: one target for all?. Psychoradiology.

[29] Kang N, Kim RK, Kim HJ (2019). Effects of transcranial direct current stimulation on symptoms of nicotine dependence: A systematic review and meta-analysis. Addict Behav.

[30] Watson NL, Carpenter MJ, Saladin ME (2010). Evidence for greater cue reactivity among low-dependent vs. high-dependent smokers. Addict Behav.

[31] Kim W-S, Paik N-J (2021). Safety Review for Clinical Application of Repetitive Transcranial Magnetic Stimulation. Brain Neurorehabilitation.

[32] Mattioli F, Maglianella V, D'Antonio S (2024). Non-invasive brain stimulation for patients and healthy subjects: Current challenges and future perspectives. J Neurol Sci.

[33] Wessel MJ, Egger P, Hummel FC (2021). Predictive models for response to non-invasive brain stimulation in stroke: A critical review of opportunities and pitfalls. Brain Stimul.

